# Disordered eating and BMI predict negative body image

**DOI:** 10.1192/j.eurpsy.2021.1222

**Published:** 2021-08-13

**Authors:** E. Fyodorova, G. Arina, V. Nikolaeva

**Affiliations:** Psychology, Lomonosov Moscow State University, Moscow, Russian Federation

**Keywords:** body image, Body Mass Index, disordered eating

## Abstract

**Introduction:**

It is known that negative body image can cause significant emotional distress for an individual and thus lead to lower subjective well-being. Previous research has shown that both disordered eating and body mass are connected to negative body image.

**Objectives:**

To examine how disordered eating and BMI can predict different aspects of body image.

**Methods:**

A sample of 180 healthy respondents (152 Female, 28 Male, mean age 22,62±7,35) were recruited in Moscow. Disordered eating was measured by Eating Attitude Test (EAT-26; Garner D. et al., 1982), body image was measured by Multidimensional Body-Self Relations Questionnaire (MBSRQ; Cash T. F., 1990). Body mass index (BMI) was calculated on the basis of self-reported data (height and weight). Multiple linear regression analysis was performed in IBM SPSS Statistics 22.0.

**Results:**

Regression model with both predictors determined self-classified weight (SCW; R^2^=0,569, p<0,0001), overweight preoccupation (OWP; R^2^=0,497, p<0,0001), body areas satisfaction (BASS; R^2^=0,259, p<0,0001), and appearance evaluation (R^2^ = 0,229, p<0,0001), but only disordered eating symptoms predicted appearance (R^2^ = 0,193, p<0,0001) and health (R^2^ = 0,036, p<0,05) orientation, and none of the predictors affected fitness or health evaluation and fitness orientation.

**Conclusions:**

Symptoms of disordered eating and body mass index in normal population can predict self-evaluation of one`s appearance as less attractive, body size as bigger and weight as heavier. Only symptoms of disordered eating predicted higher extent of investment in one’s appearance and health. And neither IBM, nor disordered eating predicted self-evaluation of one`s health and fitness or the extent of investment in fitness.

